# A case–control study of mid-pregnancy circulating cardiovascular proteins in women with subsequent preeclampsia and small for gestational age births

**DOI:** 10.1038/s41598-026-61084-7

**Published:** 2026-07-06

**Authors:** Paliz Nordlöf Callbo, Katja Junus, Emelie Lindberger, Linda Lindström, Inger Sundström Poromaa, Lina Bergman, Susanne Lager, Anna-Karin Wikström

**Affiliations:** 1 Department of Women’s and Children’s Health, Uppsala University, Uppsala, Sweden; 2 Department of Obstetrics and Gynecology, Uppsala University Hospital, Uppsala, Sweden; 3 Department of Obstetrics and Gynecology, Institute of Clinical Sciences, Sahlgrenska Academy, University of Gothenburg, Gothenburg, Sweden; 4 Department of Obstetrics and Gynaecology, Stellenbosch University, Cape Town, South Africa

**Keywords:** Angiotensin-converting enzyme 2, B-type natriuretic peptide, Cardiovascular proteins, Matrix metalloproteinase-12, Preterm preeclampsia, Small for gestational age, Term preeclampsia, Biomarkers, Cardiology, Diseases, Medical research, Physiology

## Abstract

Preeclampsia and small for gestational age (SGA) births share pathophysiologic pathways involving the cardiovascular system, inflammation, and placentation. To evaluate their pathophysiological differences and similarities, we compared alterations in mid-pregnancy circulating protein levels in women with subsequent preeclampsia and/or SGA birth. We used the Olink cardiovascular panel II to measure 92 mid-pregnancy plasma proteins in healthy pregnant women with subsequent uncomplicated pregnancies (controls; *n* = 480), term (≥ 37 weeks) preeclampsia (*n* = 233), preterm (< 37 weeks) preeclampsia (*n* = 40), SGA birth (*n* = 106), or combined preeclampsia and SGA birth (*n* = 31). False discovery rate-adjusted Kruskal–Wallis and Dunn’s post-hoc tests identified protein level differences between controls and each adverse outcome group. Compared with controls, mid-pregnancy protein alterations were most common in women with subsequent term preeclampsia, particularly involving the cardiovascular proteins angiotensin-converting enzyme 2 and B-type natriuretic peptide. Across all outcomes, matrix metalloproteinase-12 and placental growth factor were lower compared to controls, particularly in those with combined disorder. In summary, cardiovascular and inflammatory protein alternations were most common in women with subsequent preeclampsia. Shared dysregulation of matrix metalloproteinase-12 and placental growth factor, both known regulators of placentation and the vascular system, suggests their involvement in both preeclampsia and SGA births, albeit the pathways may differ.

**Supplementary Information:**

The online version contains supplementary material available at 10.1038/s41598-026-61084-7.

## Introduction

The obstetric disorders preeclampsia and fetal growth restriction jeopardize the short- and long-term health of both mother and infant^1–3^. Preeclampsia affects about 2–8 percent of all pregnancies worldwide^4^. Being born small for gestational age (SGA) is often used as a proxy for fetal growth restriction, the prevalence of which ranges from 2 to 10 percent depending on the definition^5,6^.

Preeclampsia and SGA birth often co-exist but may also present as separate disorders^3,7^. Possible pathways leading to these disorders have been previously described^8^. However, we have limited knowledge of what distinguishes these disorders in early pregnancy. Women affected by these conditions often share several predisposing risk factors, such as chronic hypertension, insulin resistance, obesity and renal disease^3,5,9,10^. Further, SGA birth and preeclampsia, specifically preterm preeclampsia (delivery < 37 gestational weeks), are associated with impaired placental remodeling occurring in the first trimester of pregnancy^3,8,11^. Conversely, term preeclampsia is more commonly associated with maternal cardiovascular, inflammatory and metabolic dysfunction^3,12–14^. Independent of shared or different features, the clinical presentation varies; some women develop preeclampsia without SGA birth with delivery before or after 37 gestational weeks, others experience SGA birth without preeclampsia, and some develop both disorders.

Exploring circulating proteins using large-scale omics data can help identify disease-associated proteins and clarify possible pathophysiological pathways. This may facilitate the search for improved prediction, prevention, and treatment. Previously, most studies included women with established disease, making it difficult to interpret whether the found alterations cause or are a consequence of the disorder.

We hypothesized that differences and similarities in pathophysiological pathways of preeclampsia subtypes (preterm and term) and SGA birth may be reflected in altered mid-pregnancy cardiovascular and inflammatory proteins compared with uncomplicated pregnancies. Therefore, the objective of this study was to explore and distinguish the pathophysiological differences and similarities in isolated preeclampsia subtypes (preterm and term), isolated SGA birth, and combined preeclampsia and SGA birth compared with uncomplicated pregnancies. By exploring 92 circulating cardiovascular and inflammatory proteins in mid-pregnancy, we aimed to clarify how early alterations in protein levels in healthy women reflect distinct and shared etiologies of these adverse pregnancy outcomes before clinical onset.

## Methods

### Recruitment of participants and blood sampling

This was a nested case–control study. The source population was pregnant women from the population-based Uppsala University Hospital Biobank of Pregnant Women (2007–2018)^15^. When attending their second-trimester routine ultrasound scan at 16–20 weeks’ gestation, women aged ≥ 18 years were asked to participate in the study. If they accepted, a venous blood sample was collected. About 97 percent of the pregnant population in Uppsala County participates in the routine ultrasound examination, and all such examinations are performed at Uppsala University Hospital^16^. Previous data show that approximately 50 percent of the pregnant population in Uppsala County donated blood samples to the biobank between 2009 and 2011^15^.

### Study population

Eligible study participants were women with singleton pregnancies who donated blood between 16 + 0 and 20 + 6 weeks of gestation and then gave birth at 22 + 0 weeks of gestation or later. Due to the explorative objective of the study, we preferred to have a homogeneous population by excluding women with a history of chronic hypertension or renal disease, ongoing treatment with immune- or coagulation-modulating medication (including aspirin) or lithium, pre-gestational or gestational diabetes, pregnancy complications including isolated proteinuria, cholestasis of pregnancy, polyhydramnios, oligohydramnios, erythrocyte alloimmunization, maternal thromboembolism, or placental abruption. This information and further clinical and demographic data on the women (maternal age, body mass index (BMI), parity, smoking status, systolic and diastolic blood pressure at first antenatal visit, gestational age at blood sampling, gestational length at birth, infant birth weight and sex) were collected from electronic medical records.

### Outcome groups

Preeclampsia was identified by International Classification of Diseases (ICD) codes (O14, O15), recorded by the responsible doctor at discharge after delivery. During the study period, preeclampsia was defined as new-onset hypertension (systolic blood pressure ≥ 140 mmHg and/or diastolic blood pressure ≥ 90 mmHg) measured at two subsequent occasions at least 4 h apart in combination with proteinuria (≥ 300 mg/24 h or a spot urine protein/creatinine ratio ≥ 30 mg/mmol or at least 1 g/L (2 +) on a dipstick test) after 20 weeks of gestation^17^. SGA birth was used as a proxy for fetal growth restriction, defined as birth weight < -2 standard deviations (SD) for gestational age and sex (corresponding to the 2.3^rd^ percentile), in line with the Delphi procedure of the International Society of Ultrasound in Obstetrics & Gynecology^18,19^. Studied outcomes were the pregnancy disorders: (1) term preeclampsia (delivery < 37 + 0 gestational weeks) with non-SGA infant, (2) preterm preeclampsia (delivery ≥ 37 + 0 gestational weeks) with non-SGA infant, (3) SGA birth (without preeclampsia), and (4) combined preeclampsia (preterm or term) with SGA birth. Due to a small number of early-onset cases (*N* = 12), the study was underpowered to evaluate preeclampsia by the subtypes early- and late-onset. All medical records were reviewed to validate the diagnoses.

Controls were healthy pregnant women without a history of preeclampsia who were eligible for inclusion and with a subsequent uncomplicated pregnancy, i.e., without development of hypertension, preterm delivery (< 37 weeks’ gestation), delivery of an infant born SGA, or stillbirth. No women participated more than once in this study. Cases were matched one-to-one with controls based on first-trimester BMI and parity. After reviewing medical records for data collected on maternal, pregnancy, and infant characteristics and validation of the preeclampsia and SGA birth diagnoses, we excluded or recategorized 19 controls, 22 women with preeclampsia, and 48 women with SGA birth, as they did not meet the inclusion or diagnosis criteria (Supporting Information, figure S2). The final cohort consisted of healthy women who continued to have an uncomplicated pregnancy (controls, *n* = 480) or developed one of the studied outcomes: term preeclampsia without SGA birth (*n* = 233), preterm preeclampsia without SGA birth (*n* = 40), SGA birth without preeclampsia (*n* = 106), or preeclampsia and SGA birth (*n* = 31).

### Biochemical analyses

Plasma samples were analyzed at the Science for Life Laboratory, Uppsala, Sweden, using the explorative Olink Proseek multiplex Cardiovascular II (CVD-II) Panel. The panel contained 92 known cardiovascular and inflammatory proteins. The analytical details of the proximity extension assay (PEA) technology and the full names of the proteins are presented in the Supporting Information. Based on negative controls included in the analyses, a limit of detection (LOD) was estimated for each PEA measurement. To use all available data, we included all proteins with measured levels (even when below LOD) for each sample rather than imputing values below LOD. Data were reported as normalized protein expression (NPX) on a Log2 − scale, expressed as relative protein values, where a one-unit increase in NPX corresponds to a doubling of the protein concentration^21^. Proteins with NPX values over the upper quantification limit of the determined dynamic range and a cropped violin plot distribution indicated a signal drop due to a state of protein excess relative to the antibody probes. These proteins were excluded from the study due to the risk of misinterpretation because of falsely lowered values^22^.

### Statistical analyses

Characteristics of the study population are presented as medians and interquartile ranges (IQRs) for continuous variables and frequencies for categorical variables. Differences between groups were tested with the Kruskal–Wallis test with Dunn’s Bonferroni post-hoc test for continuous variables or Pearson’s chi-squared test for categorical variables. Protein NPX-values are reported as median and interquartile ranges (25–75%). A false discovery rate (FDR) adjusted Kruskal**–**Wallis test was applied to identify overall differences in the distribution of protein NPX-values across the five groups. For proteins demonstrating significant overall differences, Dunn’s post-hoc test with Benjamini–Hochberg FDR correction was subsequently applied to determine individual differences between each outcome group and the control group. Further, we evaluated the differential expression between each outcome group and the control group by calculating fold changes. We also identified top proteins, defined as FDR < 0.05 and a log2 fold change < -0.1 or > 0.1.

To confirm that differences in baseline characteristics did not drive our results, we conducted unadjusted and adjusted multinominal logistic regression analyses on proteins that differed between groups. The following confounders were identified by drawing and analyzing a directed acyclic graph: age, BMI, blood pressure at the first antenatal visit, smoking, and parity (Figure S1). Although the design was a matched case–control study, the model was adjusted for first trimester BMI and parity to avoid introducing bias from these factors as confounders. We also adjusted for gestational age at blood sampling, which may influence the levels of circulating proteins. Results are presented as unadjusted and adjusted odds ratios (OR and aOR) with 95 confidence intervals (CI). The logistic regression analyses were run on complete data sets. A *p*-value of < 0.05 was considered significant.

All statistical analyses were done in the Statistical Package for the Social Sciences (SPSS) Statistics 27.0 and GraphPad Prism 10.

## Results

Characteristics of the studied cohort, as well as data on the pregnancies and infants, are presented in Table 1. At the group level, cases and controls were similar with respect to maternal and pregnancy characteristics, with some exceptions. Compared to controls, BMI was lower in women with subsequent SGA and higher in those with subsequent term preeclampsia. Both systolic and diastolic blood pressure were higher at the first antenatal visit in women who later developed term preeclampsia than in controls. Pregnancies had a shorter duration in women with subsequent preeclampsia subtypes or combined preeclampsia and SGA birth than in controls.

**Table 1:** Characteristics of the study population.

Clinical variables	Controls	Term preeclampsia	Preterm preeclampsia	SGA^a^	Preeclampsia and SGA^a^
Numbers	480	233	40	106	31
Gestational weeks at sampling	18.3 (17.7–18.9)	18.4 (17.7–19.1)*	18.4 (17.6–19.1)	18.1 (17.6–18.7)	18.3 (17.6–19.0)
Maternal characteristics, mid-pregnancy					
Age, years	30.0 (27.0–33.0)	29.0 (25.0–33.0)	30.0 (25.5–34.5)	30.0 (26.0–34.0)	32.0 (29.0–35.0)^†^
Body mass index, kg/m^2^	24.3 (22.0–28.3)	25.7 (23.0–29.8)*	23.7 (22.3–25.4)	22.7 (21.1–24.9)*	24.3 (21.0–29.7)
Nulliparity	294 (61.3)	158 (67.8)	22 (55.0)	73 (68.9)	23 (74.2)
Smoker	19 (4.0)	5 (2.1)	1 (2.5)	10 (9.4)*	1 (3.2)
Systolic blood pressure, mmHg	115 (109–121)	120.0 (114.5–128.0)^†^	120.0 (110.0–127.5)	115 (109–120)	120 (110–128)
Diastolic blood pressure, mmHg	70 (65–76)	73.0 (67.0–80.0)^†^	71.0 (65.0–80.0)	70 (65–74)	70 (65–80)
Infant characteristics					
Birth weight, grams	3,618 (3,322–3,940)	3,470 (3,090–3,830)*	2,410 (1,925–2,740)^†^	2,565 (2,370–2,755)^†^	2,050 (1,533–2,446)^†^
Gestational weeks at delivery	40.1 (39.3–40.9)	36.9 (35.7–37.8)^†^	39.9 (38.7–40.7)^†^	40.0 (38.7–41.0)	36.9 (33.4–39.3)^†^
Preterm birth, n (%)	0 (0)	40 (50)	0 (0)	8 (7.5)	16 (51.6)^†^
Sex: Girl, n (%)	225 (53.1)	39 (49)	90 (47)	42 (39.6)	11 (35.5)

Data are missing on systolic (n = 3) and diastolic blood pressure (n = 3). Controls indicate healthy pregnant women with subsequent uncomplicated pregnancy. ^a^SGA indicates women with subsequent small for gestational age infants, defined as birth weight <  − 2 standard deviations for gestational age and sex. Preterm birth indicates delivery before 37 weeks’ gestation. Data are given as medians (interquartile range) for continuous variables and frequency (%) for categorical variables. Differences between groups were tested with the Mann–Whitney *U* test or the Pearson’s chi-squared test, *p ≤ 0.05, ^†^p ≤ 0.01.

### Overview of alterations in mid-pregnancy proteins

Of the 92 analyzed circulating proteins, two (pregnancy-associated plasma protein A and interleukin-27) were excluded from further analysis due to values above the determined dynamic range, which could lead to potential misinterpretation. The FDR-adjusted Kruskal–Wallis test identified 24 proteins that were differentially expressed between the five groups: controls, women with subsequent preterm preeclampsia, term preeclampsia, SGA birth, and combined preeclampsia and SGA birth (Table 2). Of these, 21 proteins differed in total when comparing each outcome group with the controls. Log2 fold changes of these proteins are presented in Supplemental Table S2.

**Table 2:** NPX-values of circulating mid-pregnancy proteins, with differences in protein values between the pregnancy outcomes term and preterm preeclampsia without SGA birth, SGA birth, and combined preeclampsia and SGA birth compared with controls.

Protein	Controls (*n* = 480)	Term preeclampsia (*n* = 233)	Preterm preeclampsia (*n* = 40)	SGA^a^ (*n* = 106)	Preeclampsia and SGA^a^ (*n* = 31)
ACE2	3.7 (3.5–4.0)	3.85 (3.49–4.24)***	3.91 (3.77–4.18)**	3.68 (3.34–4.02)	3.59 (3.37–4.39)
ADM	7.4 (7.2–7.6)	7.42 (7.23–7.62)	7.37 (7.21–7.59)	7.31 (7.04–7.46)**	7.40 (7.08–7.55)
AMBP	7.4 (7.3–7.5)	7.45 (7.35–7.55)**	7.42 (7.34–7.57)	7.41 (7.27–7.48)	7.43 (7.34–7.61)
BMP-6	3.9 (3.5–4.2)	3.85 (3.26–4.15)	3.96 (3.53–4.20)	4.04 (3.68–4.26)*	4.03 (3.65–4.32)
BNP	1.2 (0.8–1.7)	0.95 (0.69–1.25)***	1.02 (0.60–1.26)*	1.25 (0.84–1.56)	1.20 (0.67–1.65)
CA5A	1.8 (1.4–2.3)	2.17 (1.44–2.71)**	1.94 (1.45–2.41)	1.76 (1.30–2.55)	1.79 (1.28–2.23)
HAOX1	4.2 (3.4–5.1)	4.66 (3.71–5.69)**	4.34 (3.52–5.40)	4.09 (3.17–5.23)	4.13 (3.56–4.64)
IDUA	5.9 (5.7–6.2)	6.06 (5.81–6.35)***	6.00 (5.77–6.22)	5.96 (5.60–6.32)	6.08 (5.75–6.36)
IL16	5.6 (5.4–5.9)	5.67 (5.41–5.91)	5.67 (5.36–6.03)	5.65 (5.36–5.92)	5.93 (5.70–6.10)**
IL-1ra	4.2 (3.9–4.5)	4.38 (4.07–4.69)***	4.25 (4.11–4.53)	4.19 (3.75–4.49)	4.24 (3.97–4.53)
IL-4RA	2.2 (2.0–2.3)	2.22 (2.06–2.40)**	2.15 (1.99–2.37)	2.15 (1.95–2.31)	2.18 (1.97–2.37)
KIM1	6.2 (5.9–6.6)	6.36 (6.06–6.74)**	6.41 (5.81–6.69)	6.10 (5.73–6.56)	6.20 (5.77–6.68)
Leptin	7.5 (7.0–7.9)	7.69 (7.38–7.96)**	7.79 (7.06–8.04)	7.28 (6.50–7.78)*	7.60 (6.89–8.06)
LPL	9.5 (9.2–9.8)	9.41 (9.15–9.67)*	9.44 (9.11–9.68)	9.52 (9.23–9.76)	9.66 (9.29–9.90)
MARCO	6.2 (6.0–6.3)	6.25 (6.10–6.36)**	6.25 (6.13–6.39)	6.16 (6.03–6.33)	6.22 (6.11–6.41)
MMP12	7.0 (6.7–7.4)	6.81 (6.47–7.22)***	6.62 (6.20–7.40)***	6.72 (6.29–7.24)***	6.21 (5.93–6.56)***
PAR-1	8.3 (8.0–8.5)	8.30 (8.05–8.57)	8.19 (8.04–8.45)	8.24 (8.03–8.47)	8.48 (8.24–8.77)**
PD-L2	3.3 (3.1–3.5)	3.25 (3.06–3.46)	3.48 (3.29–3.58)**	3.25 (3.06–3.51)	3.21 (3.10–3.35)
PlGF	10.0 (9.7–10.4)	9.90 (9.52–10.25)**	9.83 (9.34–10.25)*	9.84 (9.36–10.24)***	9.48 (8.67–9.89)***
PRSS8	8.7 (8.6–8.8)	8.72 (8.55–8.86)	8.78 (8.64–8.91)**	8.64 (8.50–8.76)**	8.60 (8.43–8.82)
PSGL-1	3.7 (3.6–3.8)	3.72 (3.61–3.86)	3.74 (3.62–3.85)	3.66 (3.54–3.77)	3.77 (3.58–3.92)*
SORT1	8.1 (8.0–8.3)	8.16 (7.99–8.34)**	8.19 (8.01–8.32)	8.08 (7.91–8.25)	8.19 (7.95–8.46)
TNFRSF11	5.2 (5.0–5.4)**	5.28 (5.10–5.52)	5.24 (5.07–5.48)	5.15 (4.97–5.40)	5.35 (5.12–5.48)
TRAIL-R2	5.0 (4.9–5.2)*	5.06 (4.90–5.25)	5.07 (4.92–5.16)	4.99 (4.84–5.15)	5.14 (5.00–5.25)

Data presenting overall protein differences between the outcomes identified by the false discovery rate-adjusted Kruskal–Wallis test. Protein values are given as normalized protein expression, presented by median and inter quartile ranges (25–75%). Dunn’s Bonferroni post-hoc test was applied to determine differences between the outcomes. *P < 0.05, **P < 0.01, ***P < 0.001 using controls as reference group. Controls indicates healthy pregnant women with subsequent uncomplicated pregnancy. ^a^SGA indicates women with subsequent small for gestational age infants defined as birth weight <  − 2 standard deviations for gestational age and sex.

### Outcome-specific protein alterations

**Fig. 1:**
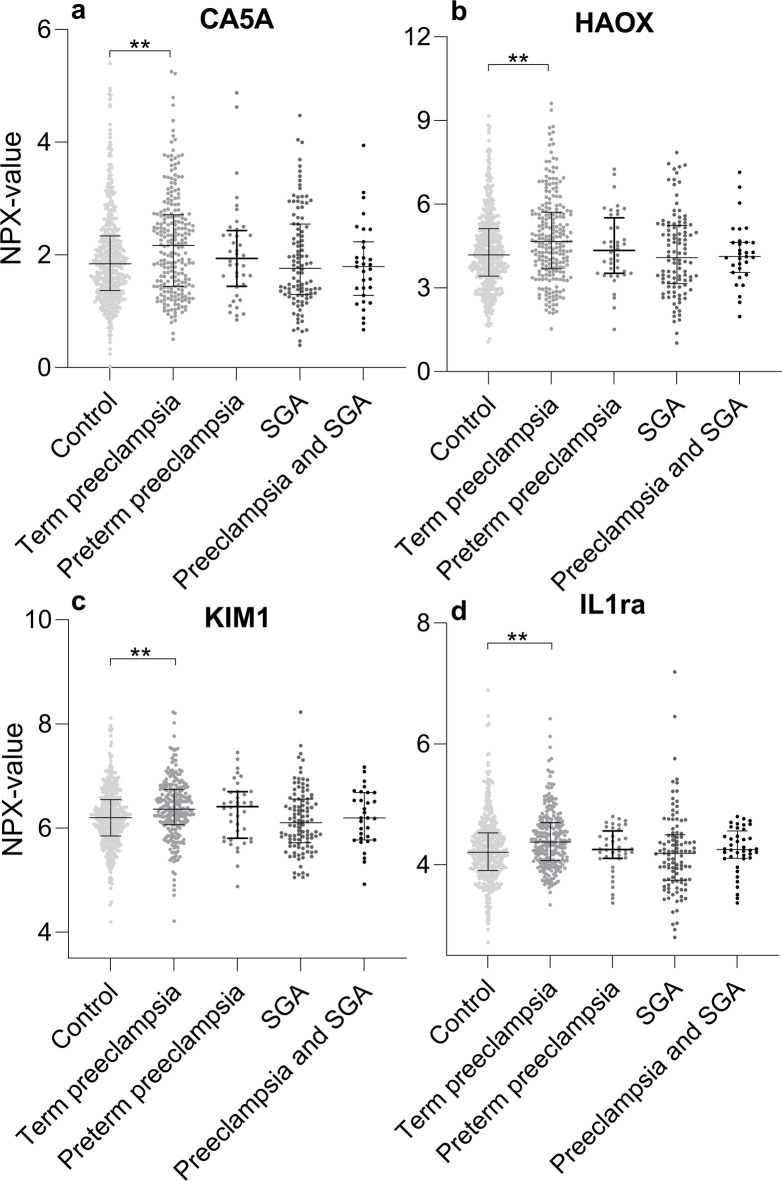
Scatterplots of the top proteins (CA5A, HAOX1, Leptin, IL1RA) with differential circulating levels in women with subsequent term preeclampsia, compared with controls. Only four of the top proteins, defined as FDR < 0.05 and fold change < − 0.1 or > 0.1, are presented. Data are given as normalized protein expression (NPX) log2 values. Horizontal bars represent the median ± interquartile range. **Indicates p < 0.01 and ***p < 0.001. SGA indicates birth of small for gestational age infant, defined as birth weight <  − 2 standard deviations for gestational age and sex. CA5A, Carbonic anhydrase 5A, mitochondrial; HAOX1, Hydroxyacid oxidase 1; IL1RA, Interleukin-1 receptor antagonist protein.

In women with subsequent term preeclampsia, 11 proteins were higher compared with controls: Alpha-1-microglobulin/bikunin precursor (AMBP), Carbonic anhydrase 5A, mitochondrial (CA5A), hydroxyacid oxidase 1 (HAOX1), alpha-L-iduronidase (IDUA), interleukin-1 receptor antagonist (IL-1RA), Interleukin-4 receptor subunit alpha (IL4RA), kidney injury molecule-1 (KIM1), Lipoprotein lipase (LPL), Macrophage receptor with collagenous structure (MARCO), tumor necrosis factor receptor superfamily member 11A (TNFRSF11A), and TNF-related apoptosis-inducing ligand receptor 2 (TRAIL-R2). In this comparison, we identified 8 top proteins (fold change < -0.1 or > 0.1): CA5A, HAOX, KIM1, IL1ra, IDUA, LPL TNFRSF11A, TRAIL-R2 (the first four presented in Fig. 1).

**Fig. 2:**
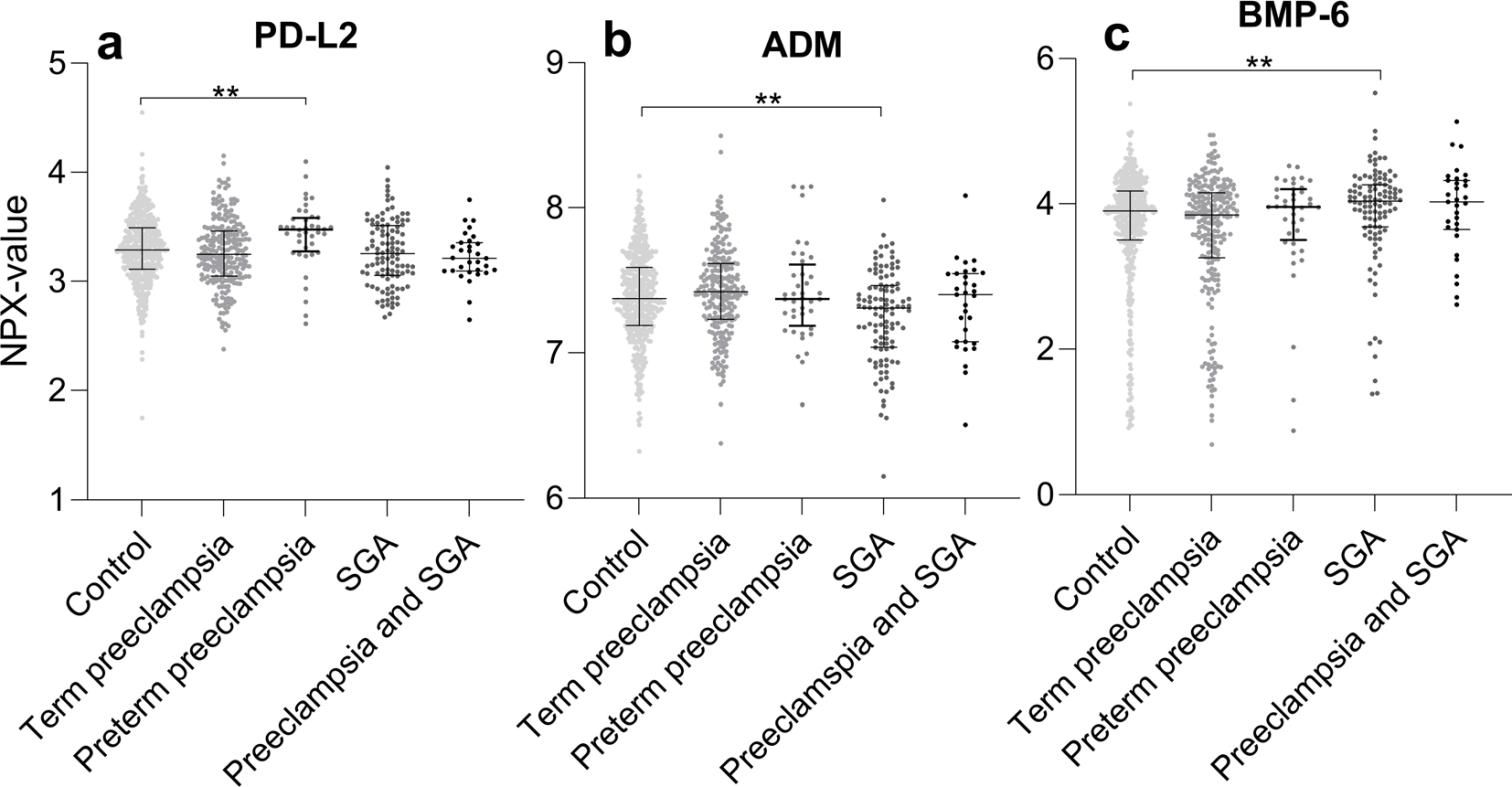
Scatterplots of the proteins with differential circulating levels in women with subsequent preterm preeclampsia (**a**), respectively SGA birth (**b**-**c**), compared with controls. Only top proteins, defined as FDR < 0.05 and fold change < − 0.1 or > 0.1, are presented. Section a: Scatterplots showing PD-L2 with differential circulating levels in women with subsequent preeclampsia, compared with controls. Section **b**-**c**: Scatterplots showing ADM and BMP-6 that were differently expressed in women with subsequent combined preeclampsia and SGA birth, compared with controls. Data are given as normalized protein expression (NPX) log2 values. Horizontal bars represent the median ± interquartile range. **Indicates p < 0.01; ***p < 0.001. SGA indicates birth of small for gestational age infant, defined as birth weight <  − 2 standard deviations for gestational age and sex. PD-L2 indicates Programmed cell death 1 ligand 2; ADM, Adrenomedullin; BMP-6, Bone morphogenetic protein 6.

Among women with subsequent preterm preeclampsia, only Programmed cell death 1 ligand 2 (PD-L2) was altered with a lower NPX-value compared with controls. PD-L2 was identified as a top protein (fold change < -0.1 or > 0.1), Fig. 2a.

In women with subsequent SGA birth, three proteins were altered compared with controls: Adrenomedullin (ADM), Bone morphogenetic protein 6 (BMP6) and P-selectin glycoprotein ligand 1 (PSGL1). ADM and PSGL1 had lower NPX-values, and BMP6 had higher NPX-values in women with subsequent SGA birth. Only ADM and BMP6 were identified as top proteins (fold change < − 0.1 or > 0.1), Fig. 2b-c.

**Fig. 3:**
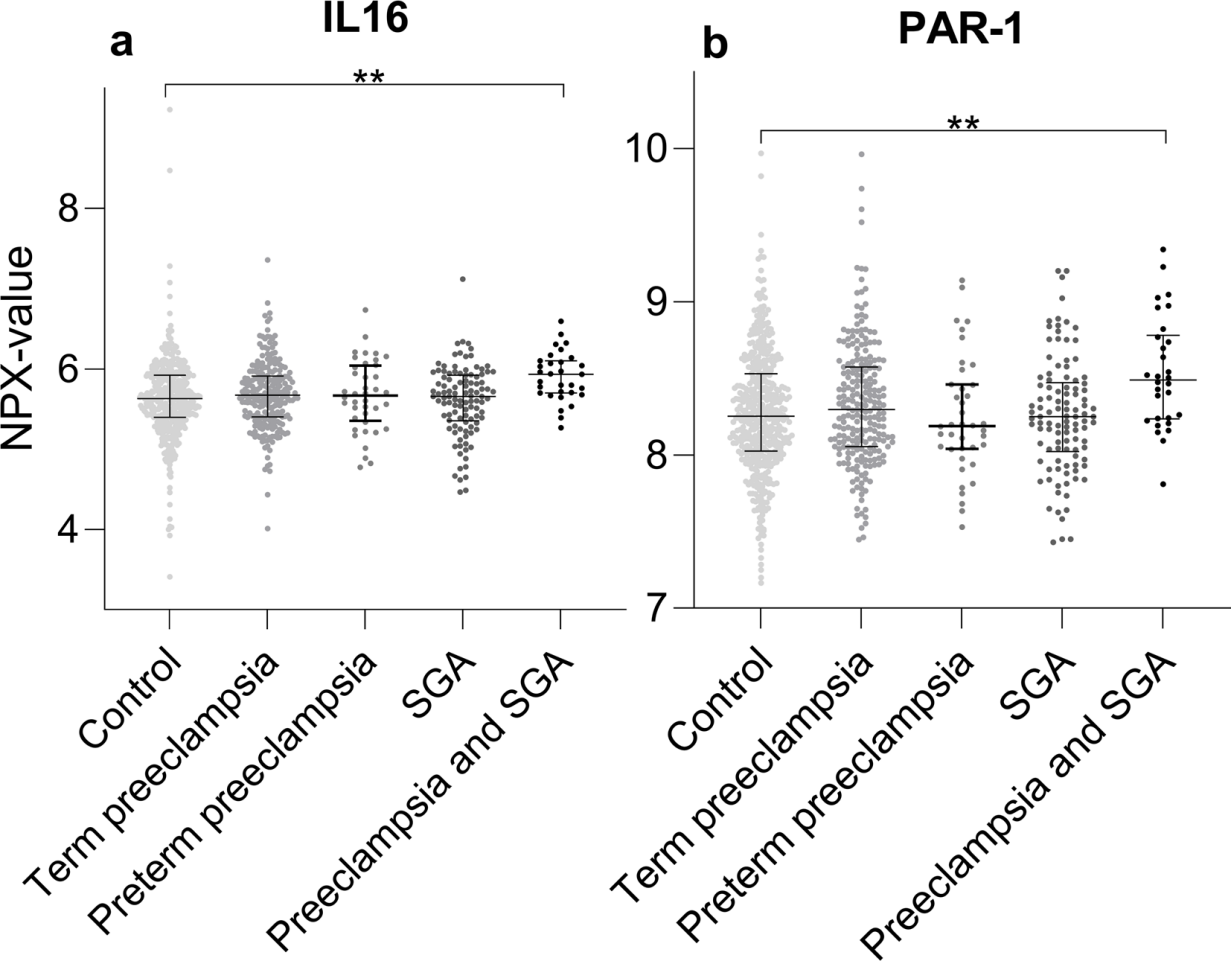
Scatterplots showing proteins (IL-16 and PAR-1) with differential circulating protein levels in women with subsequent combined preeclampsia and SGA birth, compared to controls. Only top proteins, defined as FDR < 0.05 and fold change < − 0.1 or > 0.1 are presented. Data are given as normalized protein expression (NPX) log2 values. Horizontal bars represent the median ± interquartile range. **Indicates p < 0.01; ***p < 0.001. IL-1 indicates Interleukin 16; PAR-1, Proteinase-activated receptor 1.

In women with subsequent combined preeclampsia and SGA birth, two proteins (Interleukin-16 (IL16) and Proteinase-activated receptor 1 (PAR-1)) were higher compared with controls. Both proteins were also identified as top proteins (fold change < − 0.1 or > 0.1), Fig. 3.

### Shared protein alterations

**Fig. 4:**
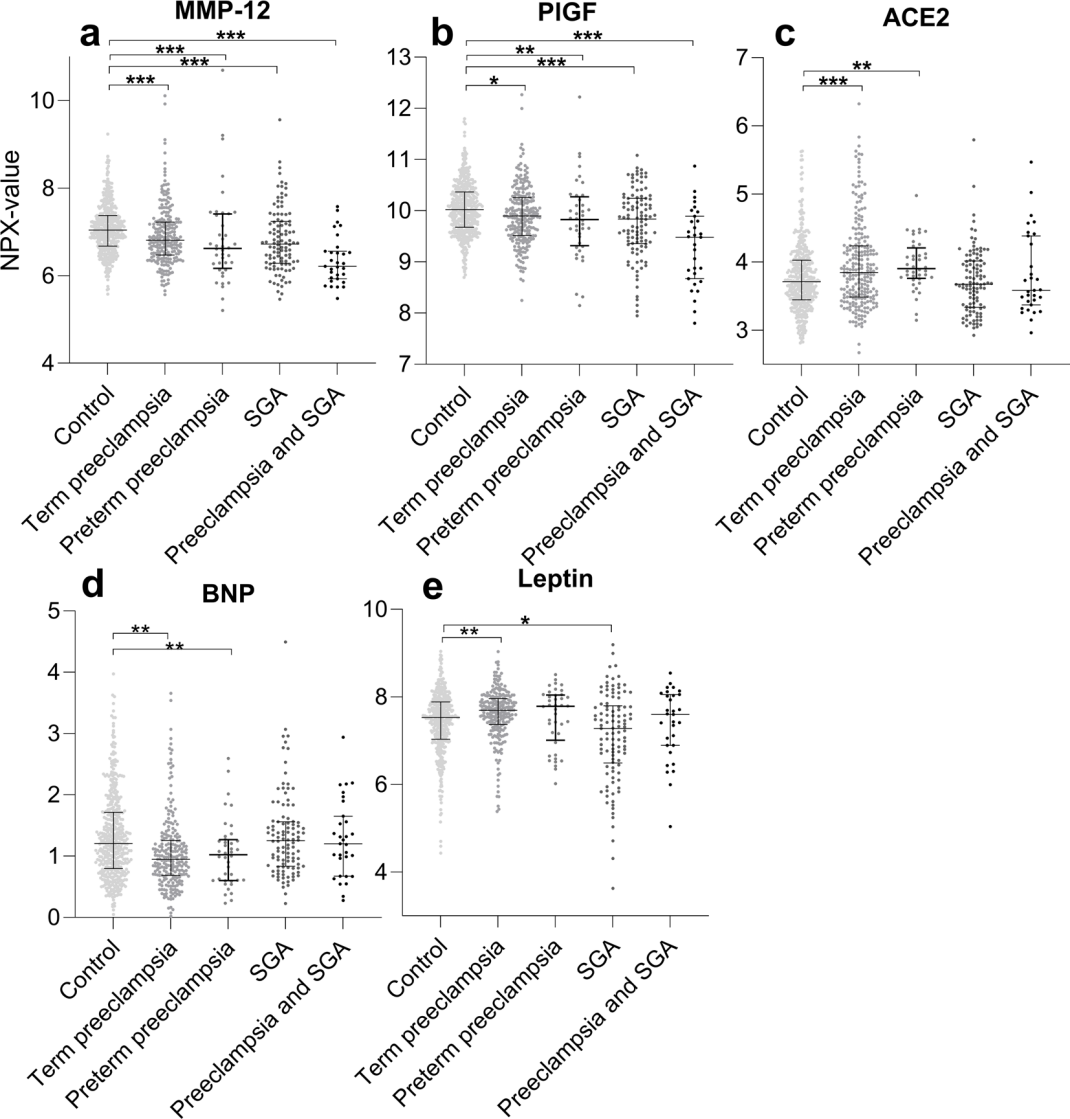
Scatterplots showing proteins with shared alterations in women with subsequent preeclampsia, SGA birth, or combined preeclampsia and SGA birth, compared to controls. Only top proteins, defined as FDR < 0.05 and fold change < − 0.1 or > 0.1 are presented. Data are given as normalized protein expression (NPX) log2 values. Horizontal bars represent the median ± interquartile range. **Indicates p < 0.01; ***p < 0.001. MMP-12 indicates matrix metalloproteinase-12; PlGF, placental growth factor, ACE2, Angiotensin-converting enzyme 2; BNP; Natriuretic peptide B.

We identified several overlapping protein alterations across the different adverse pregnancy outcome groups compared with controls. NPX-values of matrix metalloproteinase-12 (MMP-12) and placental growth factor (PlGF) were consistently lower across all four outcome groups, with the lowest values observed in the combined preeclampsia and SGA birth (Table 2). Within the preeclampsia subtypes, ACE2 expression was higher than in controls, with the most pronounced increase in the preterm group. Conversely, BNP levels were lower in preeclampsia subtypes compared with controls, with the lowest values in the term group. Leptin levels were altered with higher NPX-values in both the term preeclampsia and SGA birth groups. Prostasin (PRSS8) was altered with higher NPX-values in women with subsequent preterm preeclampsia and lower in the SGA birth group. Among these proteins, MMP-12, PlGF, ACE2, BNP and Leptin were identified as top proteins (fold change < -0.1 or > 0.1) in their respective comparisons (Fig. 4). PRSS8 was only identified as a top protein in women with subsequent preterm preeclampsia.

### Circulating proteins and their associations with pregnancy disorders

After correcting for possible confounding factors through logistic regression modelling, the associations between the 24 circulating mid-pregnancy proteins and adverse pregnancy outcomes remained unchanged for the majority of proteins. However, the associations between SGA birth with CA5A and BMP6 did not remain after adjustments. Results are presented with unadjusted and adjusted odds ratios in Supplemental Table 3. MMP-12 and PlGF NPX-values were associated with all four adverse pregnancy outcomes. Their strongest association were with the combined disorder (aOR [95% CI] for MMP-12 0.11 [0.05–0.23] and for PlGF 0.10 [0.05–0.19], both *p* < 0.001).

## Discussion

In this prospective proteomic study, we identified distinct mid-pregnancy cardiovascular and inflammatory protein alterations in women with subsequent term or preterm preeclampsia without SGA birth, SGA birth without preeclampsia and combined disorder. Notably, women with subsequent term preeclampsia presented with a multisystem dysregulation of cardiovascular and inflammatory proteins two months before clinical onset. This systemic pattern was less pronounced or absent in women with subsequent preterm preeclampsia, SGA birth, or combined disorders. However, all outcomes showed dysregulation of MMP-12 and PlGF, both known regulators of vascular function, inflammation and placental development.

Suboptimal cardiovascular features, such as elevated blood pressure, impaired cardiac output, and enhanced systemic inflammation are common risk factors for cardiovascular disease and preeclampsia^23^. In our study, women with subsequent term preeclampsia without SGA birth displayed altered expression of several cardiovascular and inflammatory proteins, including higher ACE2 and lower BNP levels than controls. This aligns with our previous findings and with other studies showing lower mid-pregnancy BNP values in women with subsequent preeclampsia^20,24,25^. The results contrast with the pattern in uncomplicated pregnancies, in which elevated BNP values in early pregnancy reflect a healthy hemodynamic response to first trimester volume expansion^26^. Conversely, ACE2 is the counter-regulatory component of the renin-angiotensin system (RAAS). Under normal conditions, BNP antagonizes the effects of RAAS, promoting vasodilation^27,28^. The mid-pregnancy imbalance of ACE2 and BNP observed in term as well as to some extent in preterm preeclampsia suggests different degrees of underlying RAAS dysregulation and altered hemodynamics, indicating a maternal cardiovascular involvement in the pathogenesis of both preeclampsia subtypes.

The maternal protein profile in women with subsequent term preeclampsia was characterized by dysregulation of both cardiovascular and inflammatory proteins. This pronounced dysregulation may reflect an underlying maternal vulnerability with increased responsiveness to syncytiotrophoblast stress and placental debris, driving the development of maternal symptoms^8^. Consequently, reducing these unfavorable maternal features prior to or during pregnancy could mitigate the risk of term preeclampsia. In contrast, women with subsequent preterm preeclampsia showed much more limited cardiovascular and inflammatory protein dysregulation. Because broad systemic dysregulation was less apparent at mid-pregnancy in women with subsequent preterm preeclampsia, pathways involving placental dysfunction may be more dominant. Similarly, cardiovascular and inflammatory dysregulation in women with subsequent SGA births was less pronounced compared to controls^3,12^. This lack of major mid-pregnancy systemic dysregulation may prevent them from developing maternal symptoms of preeclampsia^29,30^. Despite the smaller sample size in the combined preeclampsia and SGA birth group, we identified distinct alterations in the vascular and inflammatory proteins IL-16 and PAR-1, known regulators of vascular function^29–31^. These distinct protein alterations may contribute to adverse cardiovascular and inflammatory regulation, ultimately leading to both fetal growth restriction and maternal organ dysfunction.

While the extent of cardiovascular and inflammatory profiles seems to vary across the adverse pregnancy outcomes, MMP-12 dysregulation overlapped across all outcomes. Matrix metalloproteinases (MMPs) are critical for trophoblast invasion of the spiral arteries as well as for tissue and vascular remodeling and repair^32,33^. We previously identified MMP-12 as a possible early predictor for preeclampsia^20^, using the same preeclampsia cohort (defined as early- and late onset), however in that study SGA births were not differentiated from preeclampsia. Compared with controls, dysregulation of MMP-12 was most pronounced in women with subsequent preterm preeclampsia or combined preeclampsia and SGA birth. Similarly, changes in PlGF expression were observed across all outcomes. PlGF is an angiogenic factor predominantly expressed in the placenta, regulating spiral artery remodeling and is a marker of general endothelial dysfunction^34^. It is a well-known early- and mid-pregnancy marker for preterm preeclampsia and SGA birth^35–37^. PlGF alterations were most pronounced in women with subsequent preterm preeclampsia, SGA birth or combined preeclampsia and SGA birth, possibly contributing to both mal-placentation and systemic vascular and inflammatory dysregulation. Further, the less pronounced reduction of PlGF in term preeclampsia, may reflect PlGFs role in a general dysregulation of systemic vascular and inflammatory dysregulation rather than primary mal-placentation.

The results of this study allow exploration of the pathways that separate and unite preeclampsia subtypes and SGA birth, within the framework of the revised two-stage model^8^. It has been proposed that placental stress releases antiangiogenic factors into the maternal circulation, driving excessive vascular inflammation^8^. We suggest that women with subsequent isolated SGA birth have effective compensatory mechanisms or a resilient vascular system, allowing them to tolerate these angiogenic factors without developing preeclampsia. In contrast, women who develop term preeclampsia may present with a pre-existing multisystem vulnerability. When exposed to vascular stress due to cardiovascular and inflammatory changes of pregnancy, this underlying fragility triggers an exaggerated response leading to clinical maternal symptoms of preeclampsia. Nevertheless, in terms of both pronounced mal-placentation and multiorgan dysregulation, excessive vascular inflammation following syncytiotrophoblast stress may result in preterm preeclampsia or combined preeclampsia and SGA birth. However, our sample size may have masked the extent of these systemic differences.

A major strength of this study is the large sample size from a population-based cohort, enabling exploration of cases of preterm and term preeclampsia and SGA birth, as well as a study group of combined preeclampsia and SGA birth. Further, the data on the study population were prospectively collected. The exclusion criteria ensured homogeneity and a healthy population, enabling exploration of protein differences that may have been masked in a more inclusive cohort. Further, the study design enables exploration of pathophysiological differences in women without strong risk factors for preeclampsia or SGA birth who still develop the disorders. Blood sampling in the first half of pregnancy (~ 18 weeks’ gestation) enabled exploration of protein levels and potential pathophysiological pathways before development of clinical symptoms. However, due to inclusion of healthy women in the biobank and the strict validation of preeclampsia diagnoses, the number of women with subsequent preeclampsia during the study period was lower than reported in national registries. One potential limitation is the relative protein values generated by the Olink Proseek multiplex CVD-II Panel. However, a previous study using a similar Olink multiplex CVD assay showed that the correlation between relative and absolute PlGF protein values was excellent^38^. Another limitation is the restricted number of participants with preterm preeclampsia and the combined outcome of preeclampsia and SGA birth. Further, because biobank participants were more likely to be born in Sweden, to have more than 12 years of education, and to smoke less than non-participants, the generalizability of our findings may be limited to similar populations^39^.

## Conclusion

Our findings support heterogeneous origins of the adverse pregnancy outcomes studied, where term preeclampsia predominantly presents as a maternal multisystem disorder with mid-pregnancy cardiovascular and inflammatory dysregulation. Conversely, women with subsequent SGA birth seem to lack this dysregulation. Overlapping dysregulation of key vascular and placental proteins, MMP-12 and PlGF, highlights a shared pathway across all outcomes, although the pathophysiological mechanisms may differ. Future research should prioritize clarifying these pathways to optimize cardiovascular and inflammatory regulation as well as placentation in early pregnancy.

## Supplementary Information

Below is the link to the electronic supplementary material.

Supplementary Material 1

## Data Availability

The anonymized data supporting the findings of this study are available from the corresponding author upon request. However, access is restricted due to the sensitive nature of the data set, ongoing studies, and limitations in ethical approval (including participant informed consent).
