# Supplementary material for: A case–control study of mid-pregnancy circulating cardiovascular proteins in women with subsequent preeclampsia and small for gestational age births
Source: Sci Rep. 2026 Jul 6;16:20767. doi: 10.1038/s41598-026-61084-7 (PMC13338029; doi:10.1038/s41598-026-61084-7)
Supplement: Supplementary file 1 — Supplementary Material 1 [file 41598_2026_61084_MOESM1_ESM.docx]

Supplementary Information

Article title

A case-control study of mid-pregnancy circulating cardiovascular proteins in women with subsequent preeclampsia and small for gestational age births

Names of the authors and affiliations

Paliz Nordlöf Callbo^1, 2^, M.D.; Katja Junus^1, 2^, M.D., Ph.D.; Emelie Lindberger^1^, M.D., Ph.D.; Linda Lindström^1, 2^, M.D., Ph.D.; Inger Sundström Poromaa ^1, 2^, M.D., Ph.D.; Lina Bergman^1, 3, 4^, M.D., Ph.D.; Susanne Lager^1^, M.Sc., Ph.D.; Anna-Karin Wikström^1,2^, M.D., Ph.D.

1. Department of Women’s and Children’s Health, Uppsala University, Sweden
2. Department of Obstetrics and Gynaecology, Uppsala University Hospital, Uppsala, Sweden
3. Department of Obstetrics and Gynaecology, Institute of Clinical Sciences, Sahlgrenska Academy, University of Gothenburg, Gothenburg, Sweden
4. Department of Obstetrics and Gynaecology, Stellenbosch University, Cape Town, South Africa

Corresponding author

Paliz Nordlöf Callbo

Department of Women’s and Children’s Health, Uppsala University, Sweden

Akademiska sjukhuset, SE 751 85 Uppsala, Sweden

Telephone number: 0046700913986; e-mail: Paliz.Nordlof_Callbo@kbh.uu.se

ORCID: 0000-0002-4308-3745

Expanded materials and methods

*Biochemical analyses*

The inclusion rate depended on the availability of the research nurse and on the participant’s acceptance, where lack of time and fear of needles were the most common reasons for the decline. The women included in this Biobank represent a population of varied ethnic and socioeconomic backgrounds and had no blood-borne diseases caused by viruses or bacteria. Aspirin prophylaxis for preeclampsia prevention was selectively used in Sweden during this time, with approximately 1-2% of nulliparous women receiving treatment.^1^ Plasma samples were centrifuged (1,500g for 10 min) and stored at -70°C within two hours after sampling. The Olink’s Proseek multiplex Cardiovascular II (CVD-II) Panel uses Proximity extension assay (PEA) technology, followed by quantification using real-time PCR on the Fluidigm BioMark HD real-time PCR platform.^2^ Olink® Target 96 supplies this high through-put multiplex immunoassay to measure 92 proteins across 96 samples simultaneously, using one microliter of plasma. The proximity extension assay, allows antibodies to pairwise bind to the target protein in the sample. A proximity-dependent DNA polymerization event produces a new target sequence for real-time polymerase chain reaction (PCR). This sequence is detected and quantified using standard PCR. Each assay plate included cases, interplate controls (allowing for adjustments of any differences between runs) and negative controls. The limit of detection (LOD) is based on the background plus three standard deviations estimated from negative controls and calculated separately for each sample plate. We included all proteins with the actual measured level (even when below LOD) for each sample instead of imputing values below LOD.


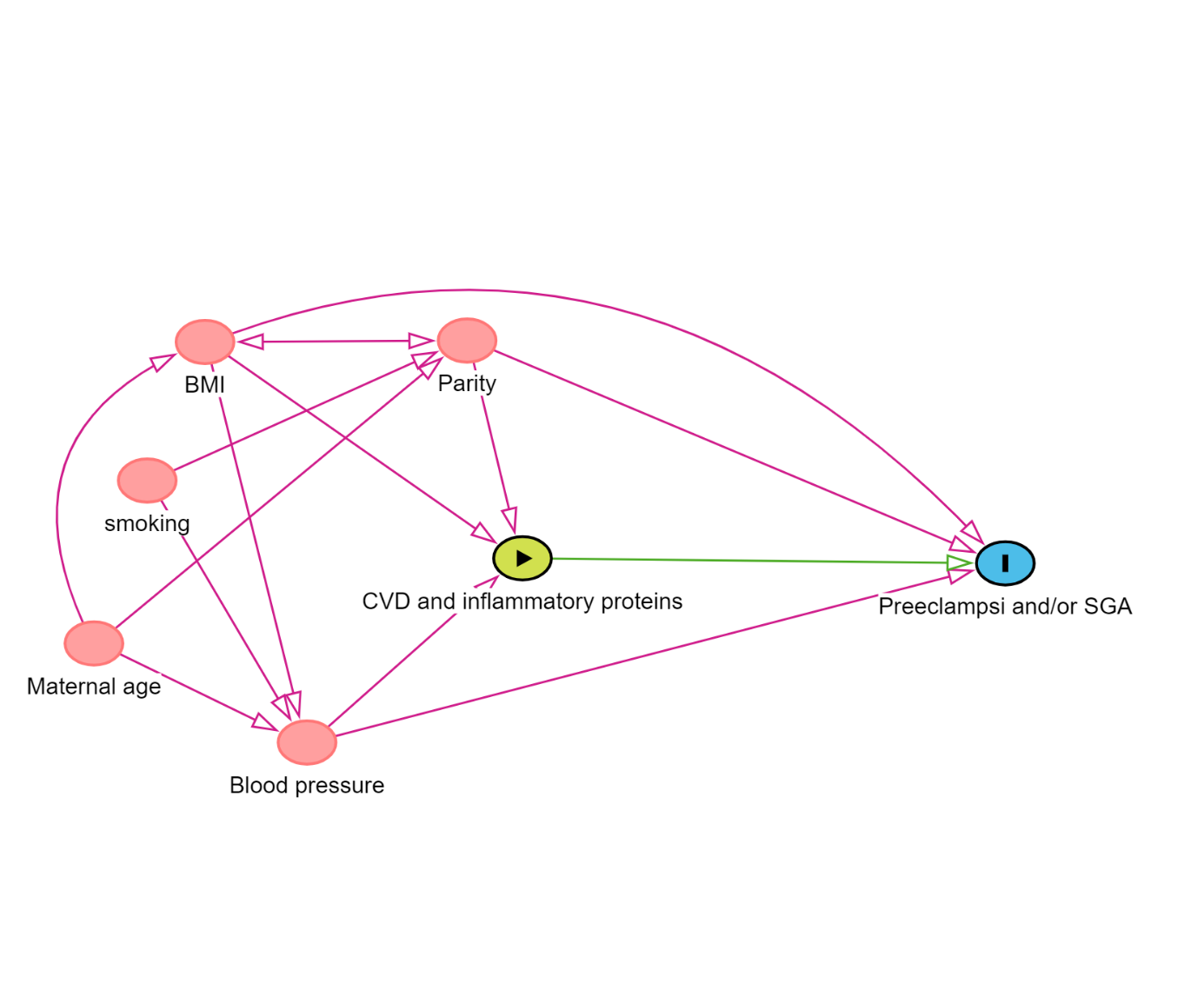


**Fig. S1** Directed acyclic graph illustrating the causal effect of cardiovascular and inflammatory proteins on preeclampsia, SGA and preeclampsia and SGA. Variables that could affect both the cardiovascular and inflammatory proteins (exposure) and the outcomes preeclampsia, SGA birth or preeclampsia and SGA birth, were identified as confounders: age, body mass index, blood pressure at the first antenatal visit, smoking, and parity. Colour coding of squares: green=exposure, blue=outcome, pink=confounder. Colour coding of arrows: green= casual path, pink=biasing path. BMI indicates body mass index. SGA indicates birth of small-for-gestational-age infant, defined as birth weight <−2 standard deviations for gestational age and sex.


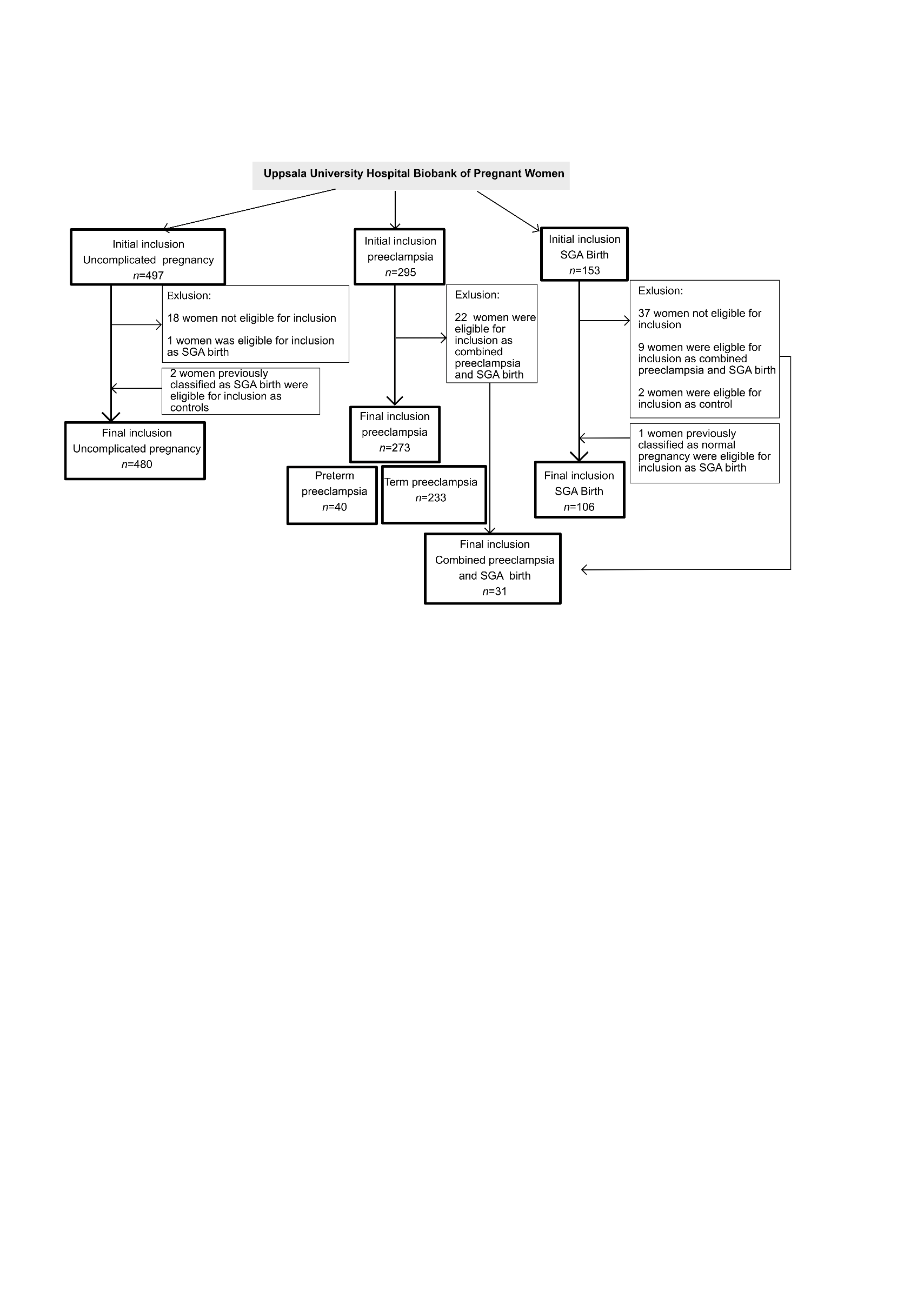


**Fig. S2** Flow chart of the study population. Cases and controls were extracted from the Uppsala University Hospital Biobank of Pregnant Women. After reviewing medical records for data collected on maternal, pregnancy, and infant characteristics as well as validation of the preeclampsia and SGA birth diagnoses some cases and controls were not eligible for inclusion and thus excluded. Some cases and controls were recategorized. SGA indicates birth of small-for-gestational-age infant, defined as birth weight <−2 standard deviations for gestational age and sex.

**Table S1.** Biomarkers in Proseek Multiplex Cardiovascular II panel

| Abbreviation | Full name |
| --- | --- |
| ACE2 | Angiotensin-converting enzyme 2 |
| ADAM-TS13 | A disintegrin and metalloproteinase with thrombospondin 13 |
| ADM | Adrenomedullin |
| AGRP | Agouti-related protein |
| AMBP | Protein AMBP |
| ANGPT1 | Angiopoietin-1 |
| BMI | Body mass index |
| BMP-6 | Bone morphogenetic protein 6 |
| BNP | Natriuretic peptide B |
| BOC | Brother of CDO |
| CA5A | Carbonic anhydrase 5A, mitochondrial |
| CCL17 | C-C motif chemokine 17 |
| CCL3 | C-C motif chemokine 3 |
| CD4 | T-cell surface glycoprotein CD4 |
| CD40-L | CD40 ligand |
| CD84 | SLAM family member 5 |
| CEACAM8 | Carcinoembryonic antigenrelated cell adhesion molecule 8 |
| CTRC | Chymotrypsin C |
| CTSL1 | Cathepsin L1 |
| CXCL1 | C-X-C motif chemokine 1 |
| DCN | Decorin |
| DECR1 | 2,4-dienoyl-CoA reductase, mitochondrial |
| Dkk-1 | Dickkopf-related protein 1 |
| FABP2 | Fatty acid-binding protein, intestinal |
| FGF21 | Fibroblast growth factor 21 |
| FGF-23 | Fibroblast growth factor 23 |
| FS | Follistatin |
| Gal-9 | Galectin-9 |
| GDF-2 | Growth/differentiation factor 2 |
| GH | Growth hormone |
| GIF | Gastric intrinsic factor |
| GLO1 | Lactoylglutathione lyase |
| GT | Gastrotropin |
| HAOX1 | Hydroxyacid oxidase 1 |
| HB-EGF | Proheparin-binding EGF-like growth factor |
| HO-1 | Heme oxygenase 1 |
| hOSCAR | Osteoclast-associated immunoglobulin-like receptor |
| HSP 27 | Heat shock 27 kDa protein |
| IDUA | Alpha-L-iduronidase |
| IgG Fc receptor II-b | Low affinity immunoglobulin gamma Fc region receptor II-b |
| IL16 | Pro-interleukin-16 |
| IL-17D | Interleukin-17D |
| IL-18 | Interleukin-18 |
| IL-1ra | Interleukin-1 receptor antagonist protein |
| IL1RL2 | Interleukin-1 receptor-like 2 |
| IL-27 | Interleukin-27 |
| IL-4RA | Interleukin-4 receptor subunit alpha |
| IL6 | Interleukin-6 |
| ITGB1BP2 | Melusin |
| KIM1 | Kidney Injury Molecule |
| LEP | Leptin |
| LOX-1 | Lectin-like oxidized LDL receptor 1 |
| LPL | Lipoprotein lipase |
| MARCO | Macrophage receptor with collagenous structure |
| MERTK | Tyrosine-protein kinase Mer |
| MMP-12 | Matrix metalloproteinase-12 |
| MMP-7 | Matrix metalloproteinase-7 |
| NEMO | NF-kappa-B essential modulator |
| PAPP-A | Pappalysin-1 |
| PAR-1 | Proteinase-activated receptor 1 |
| PARP-1 | Poly [ADP-ribose] polymerase 1 |
| PDGF subunit B | Platelet-derived growth factor subunit B |
| PD-L2 | Programmed cell death 1 ligand 2 |
| PGF | Placental growth factor |
| PIgR | Polymeric immunoglobulin receptor |
| PRELP | Prolargin |
| PRSS27 | Serine protease 27 |
| PRSS8 | Prostasin |
| PSGL-1 | P-selectin glycoprotein ligand 1 |
| PTX3 | Pentraxin-related protein PTX3 |
| RAGE | Receptor for advanced glycosylation end products |
| REN | Renin |
| SCF | Stem cell factor |
| SERPINA12 | Serpin A12 |
| SLAMF7 | SLAM family member 7 |
| SOD2 | Superoxide dismutase [Mn], mitochondrial |
| SORT1 | Sortilin |
| SPON2 | Spondin-2 |
| SRC | Proto-oncogene tyrosine-protein kinase Src |
| STK4 | Serine/threonine-protein kinase 4 |
| TF | Tissue factor |
| TGM2 | Protein-glutamine gamma-glutamyltransferase 2 |
| THBS2 | Thrombospondin-2 |
| THPO | Thrombopoietin |
| TIE2 | Angiopoietin-1 receptor |
| TM | Thrombomodulin |
| TNFRSF10A | Tumor necrosis factor receptor superfamily member 10A |
| TNFRSF11A | Tumor necrosis factor receptor superfamily member 11A |
| TNFRSF13B | Tumor necrosis factor receptor superfamily member 13B |
| TRAIL-R2 | TNF-related apoptosis-inducing ligand receptor 2 |
| VEGFD | Vascular endothelial growth factor D |
| VSIG2 | V-set and immunoglobulin domain-containing protein 2 |
| XCL1 | Lymphotactin |

Expanded results

**Table S2.** Log2 fold change calculated for each circulating mid-pregnancy protein identified as differentially expressed through false discovery rate adjusted Kruskal-Wallis test with a Benjamini-Hochberg FDR correction.

|  | *Term*  *preeclampsia* | *Preterm*  *preeclampsia* | *SGA birth* | *Preeclampsia*  *and SGA birth* |  |
| --- | --- | --- | --- | --- | --- |
| ACE2 | 0.20 | 0.20 | -0.05 | 0.05 |  |
| ADM | 0.04 | 0.04 | -0.13 | -0.06 |  |
| AMBP | 0.04 | 0.02 | -0.02 | 0.03 |  |
| ~~BMP-6~~ | ~~-0.10~~ | ~~0.06~~ | ~~0.20~~ | ~~0.25~~ |  |
| BNP | -0.27 | -0.28 | 0.02 | -0.05 |  |
| CA5A | 0.25 | 0.09 | -0.02 | -0.12 |  |
| HAOX1 | 0.47 | 0.09 | -0.10 | -0.11 |  |
| IDUA | 0.15 | 0.08 | 0.03 | 0.13 |  |
| IL16 | 0.04 | 0.03 | -0.03 | 0.27 |  |
| IL-1ra | 0.18 | 0.008 | -0.05 | 0.09 |  |
| IL-4RA | 0.08 | -0.004 | -0.03 | 0.03 | |
| KIM1 | 0.17 | 0.10 | -0.06 | -0.02 | |
| Leptin | 0.19 | 0.12 | -0.31 | -0.03 | |
| LPL | -0.11 | -0.07 | 0.06 | 0.08 | |
| MARCO | 0.06 | 0.05 | 0.0007 | 0.05 | |
| MMP12 | -0.16 | -0.24 | -0.26 | -0.73 | |
| PAR-1 | 0.06 | -0.04 | -0.02 | 0.25 | |
| PD-L2 | -0.02 | 0.14 | -0.03 | -0.07 | |
| PGF | -0.14 | -0.21 | -0.29 | -0.74 | |
| PRSS8 | 0.03 | 0.11 | -0.05 | -0.05 | |
| PSGL-1 | 0.02 | 0.01 | -0.05 | 0.03 | |
| TNFRSF11 | 0.11 | 0.04 | -0.03 | 0.09 | |
| TRAIL-R2 | 0.10 | 0.05 | -0.03 | 0.10 | |

**Table S3.** Odds ratios for each circulating mid-pregnancy protein identified through false discovery rate adjusted Kruskal-Wallis test with a Benjamini-Hochberg FDR correction and its association to subsequent term and preterm preeclampsia, SGA birth and combined preeclampsia and SGA birth.

| *Protein* | *Term preeclampsia* | *Preterm preeclampsia* | *Small for Gestational Age* | *Preeclampsia with Small for Gestational Age Birth* |
| --- | --- | --- | --- | --- |
| ACE2 | 1.95 (1.45-2.60)*** | 1.93 (1.11-3.37)* | 0.82 (0.53-1.27) | 1.22 (0.61-2.44)* |
|  | 1.76 (1.30-2.39)*** | 2.22 (1.22-4.05)** | 0.77 (0.47-1.25) | 0.95 (0.46-1.97) |
| ADM | 1.57 (0.94-2.62) | 1.45 (0.51-4.15) | 0.27 (0.14-0.52) *** | 0.57 (0.18-1.80) |
|  | 1.50 (0.86-2.62) | 1.49 (0.51-4.35) | 0.25 (0.13-0.51)*** | 0.52 (0.15-1.76) |
| AMBP | 4.07 (1.62-10.24)** | 2.20 (0.33-12.46) | 0.47 (0.13-1.65) | 2.68 (0.32-22.00) |
|  | 2.73 (1.03-7.24)* | 2.40 (0.35-16.57) | 0.56 (0.15-2.13) | 1.37 (0.15-12.25) |
| BMP6 | 0.87 (0.72-1.04) | 1.10 (0.73-1.68)*** | 1.46 (1.06-2.00)* | 1.67 (0.92-3.02) |
|  | 0.95 (0.78-1.15) | 1.07 (0.69-1.65) | 1.30 (0.94-1.81) | 1.70 (0.95-3.06) |
| BNP | 0.50 (0.38-0.66)*** | 0.48 (0.27-0.87)* | 1.04 (0.77-1.40) | 0.91 (0.53-1.55) |
|  | 0.55 (0.42-0.73)*** | 0.49 (0.27-0.90)* | 1.00 (0.73-1.36) | 0.91 (0.52-1.57) |
| CA5A | 1.37 (1.15-1.64)*** | 1.13 (0.78-1.65) | 0.97 (0.74-1.25) | 0.82 (0.51-1.32) |
|  | 1.30 (1.07-1.57)** | 1.25 (0.59-1.28) | 1.02 (0.78-1.33) | 0.68 (0.41-1.13) |
| HAOX1 | 1.25 (1.12-1.39)*** | 1.05 (0.84-1.32) | 0.95 (0.81-1.11) | 0.94 (0.74-1.23) |
|  | 1.21 (1.08-1.36)** | 1.11 (0.88-1.41) | 1.00 (0.85-1.18) | 0.87 (0.66-1.15) |
| IDUA | 2.17 (1.51-3.12)*** | 1.46 (0.71-3.02) | 1.15 (0.72-1.83) | 1.93 (0.84-4.46) |
|  | 2.07 (1.41-3.05)** | 1.59 (0.75-3.36) | 1.10 (0.68-1.77) | 1.90 (0.80-4.54) |
| IL16 | 1.24 (0.87-1.76) | 1.16 (0.56-2.39) | 0.84 (0.53-1.35) | 2.84 (1.49-5.44)** |
|  | 1.05 (0.72-1.54) | 1.27 (0.63-2.54) | 0.90 (0.56-1.46) | 2.70 (1.37-5.31)** |
| IL1ra | 1.79 (1.35-2.38)*** | 1.00 (0.54-1.87) | 0.82 (0.54-1.24) | 1.39 (0.72-2.68) |
|  | 1.52 (1.10-2.10)* | 1.28 (0.85-2.53) | 1.16 (0.74-1.81) | 1.31 (0.64-2.65) |
| IL-4RA | 2.68 (1.52-4.73)*** | 0.93 (0.28-3.16) | 0.69 (0.31-1.54) | 1.49 (0.40-5.59) |
|  | 2.18 (1.21-3.96)* | 0.97 (0.28-3.32) | 0.75 (0.32-1.75) | 1.20 (0.29-4.90) |
| KIM1 | 1.68 (1.27-2.22)*** | 1.39 (0.79-2.44) | 0.83 (0.57-1.21) | 0.95 (0.50-1.81) |
|  | 1.55 (1.51-2.68)** | 1.56 (0.86-2.84) | 0.86 (0.58-1.27) | 0.77 (0.40-1.48) |
| Leptin | 1.50 (1.18-1.90)*** | 1.27 (0.79-2.03) | 0.63 (0.49-0.81)*** | 0.95 (0.58-1.53) |
|  | 1.25 (0.95-1.63) | 1.74 (1.04-2.91)* | 0.74 (0.54-1.00)* | 0.84 (0.48-1.46) |
| LPL | 0.60 (0.42-0.84)** | 0.71 (0.35-1.43) | 1.37 (0.82-2.27) | 1.61 (0.66-3.96) |
|  | 0.69 (0.48-0.99)* | 0.69-0.33-1.46) | 1.17 (0.69-1.99) | 1.70 (0.68-4.20) |
| MARCO | 3.71 (1.71-7.77)*** | 2.91 (0.85-13.06) | 1.02 (0.38-2.72) | 3.02 (0.56-16.36) |
|  | 2.50 (1.14-5.47)* | 3.57 (0.78-16.49) | 1.58 (0.57-4.43) | 2.33 (0.40-13.45) |
| MMP12 | 0.69 (0.53-0.88)** | 0.55 (0.32-0.95)* | 0.53 (0.37-0.75)*** | 0.10 (0.05-0.22)*** |
|  | 0.74 (0.57-0.96)* | 0.53 (0.31-0.91)* | 0.45 (0.31-0.66)*** | 0.11 (0.05-0.23)*** |
| PAR-1 | 1.48 (1.00-2.21) | 0.75 (0.32-1.74) | 0.88 (0.51-1.52) | 4.27 (1.87-9.84)*** |
|  | 1.37 (0.90-2.68) | 0.79 (0.33-1.88) | 0.86 (0.49-1.51) | 4.06 (1.74-9.45)** |
| PD-L2 | 0.71 (0.46-1.29) | 4.57 (1.54-13.57)** | 0.76 (0.35-1.41) | 0.48 (0.15-1.56) |
|  | 0.72 (0.41-1.24) | 3.38 (1.24(11.88)* | 0.55 (0.27-1.13) | 0.40 (0.12-1.35) |
| PlGF | 0.65 (0.49-0.86)** | 0.51 (0.29-0.91)* | 0.40 (0.27-0.58)*** | 0.12 (0.06-0.22)*** |
|  | 0.60 (0.44-0.83)** | 0.42 (0.24-0.76)** | 0.33 (0.23-0.56)*** | 0.10 (0.05-0.19)*** |
| PSGL-1 | 1.65 (0.79-3.45) | 1.26 (0.28-5.69) | 0.34 (0.13-0.85) | 2.05 (0.37-11.41)* |
|  | 1.79 (0.84-3.84) | 1.10 (0.24-5.08) | 0.32 (0.12-0.85) | 2.23 (0.41-12.26)* |
| TNFRSF11A | 2.61 (1.62-4.19)*** | 1.51 (0.57-3.99) | 0.73 (0.38-1.41) | 2.17 (0.78-6.26) |
|  | 2.10 (1.27-3.47)** | 1.67 (0.62-4.50) | 0.83 (0.42-1.62) | 1.73 (0.89-5.11) |
| TRAIL-R2 | 2.88 (1.58-5.25)*** | 2.07 (0.66-6.51) | 0.57 (0.24-1.35) | 2.94 (1.26-6.83) |
|  | 2.36 (1.26-4.43) | 2.03 (0.81-5.11) | 0.52 (0.21-1.28) | 2.25 (0.90-5.66) |
| PRSS8 | 2.16 (1.02-4.61)* | 12.65 (2.64- 50.78)** | 0.30 (0.11-0.87)* | 0.33 (0.06-1.87) |
|  | 1.65 (0.74-3.70) | 10.13 (2.01-51.08)** | 0.2 (0.07-0.56)** | 0.27 (0.05-1.60) |

Multinominal logistic regression analysis showing unadjusted (first row) and adjusted (second row) odds ratio (95% confidence intervals) for each protein. Controls were used as reference category. Adjustments were made for parity, maternal age, first trimester BMI, smoking habits, first trimester systolic and diastolic blood pressure, and gestational age at blood sampling. SGA indicates small for gestational age, defined as birth weight <-2 standard deviations for the gestational age and sex. **P*≤ 0.05, ***P*≤0.01, ****P*≤0.001

References

1. Sandström A, Snowden JM, Höijer J, Bottai M, Wikström AK. Clinical risk assessment in early pregnancy for preeclampsia in nulliparous women: A population based cohort study. PLoS One. 2019;14:e0225716. doi:10.1371/journal.pone.0225716

2. Assarsson E, Lundberg M, Holmquist G, Björkesten J, Thorsen SB, Ekman D, Eriksson A, Rennel Dickens E, Ohlsson S, Edfeldt G, Andersson AC, Lindstedt P, Stenvang J, Gullberg M, Fredriksson S. Homogenous 96-plex PEA immunoassay exhibiting high sensitivity, specificity, and excellent scalability. PLoS One. 2014;9:e95192. doi:10.1371/journal.pone.0095192
